# Screening for Infectious Diseases among Newly Arrived Migrants in EU/EEA Countries—Varying Practices but Consensus on the Utility of Screening

**DOI:** 10.3390/ijerph111011004

**Published:** 2014-10-21

**Authors:** Tommi Kärki, Christian Napoli, Flavia Riccardo, Massimo Fabiani, Maria Grazia Dente, Manuel Carballo, Teymur Noori, Silvia Declich

**Affiliations:** 1 National Centre for Epidemiology, Surveillance and Health Promotion, National Institute of Health (Istituto Superiore di Sanità, ISS), viale Regina Elena, 299-00161 Rome, Italy; E-Mails: christian.napoli@iss.it (C.N.); flavia.riccardo@iss.it (F.R.); massimo.fabiani@iss.it (M.F.); mariagrazia.dente@iss.it (M.G.D.); silvia.declich@iss.it (S.D.); 2 European Programme for Intervention Epidemiology Training (EPIET), European Centre for disease Prevention and Control (ECDC), Tomtebodavägen 11a, 171 83 Stockholm, Sweden; 3 International Centre for Migration, Health and Development (ICMHD), Route du Nant d’Avril 11, CH–1214 Geneva, Switzerland; E-Mail: mcarballo@icmhd.ch; 4 European Centre for Disease Prevention and Control (ECDC), Tomtebodavägen 11a, 171 83 Stockholm, Sweden; E-Mail: teymur.noori@ecdc.europa.eu

**Keywords:** screening, immigration, refugees, migrants, infectious diseases

## Abstract

Screening is one possible tool for monitoring infectious diseases among migrants. However, there is limited information on screening programmes targeted for newly arrived migrants in EU/EEA countries. Our aim was to investigate the implementation, practices and usefulness of these programmes. We conducted a survey among country experts from EU/EEA countries and Switzerland, asking whether their countries had implemented screening programmes. We also estimated the association between the implementation of these programmes and the rate of asylum-seekers in the population. Of the countries, 16 (59%) had implemented screening programmes and 15 (56%) had national guidelines. The rate of asylum-seekers was associated with implementation of screening programmes (*p* = 0.014). Screening was performed most often for tuberculosis; most commonly on holding level, and was targeted to specific migrant groups in over half of the countries performing screening. Twenty-five of all the country experts (96%) considered screening among migrants useful, and 24 (92%) would welcome EU level guidelines for screening. The implementation of screening programmes varied, and the practices were different among countries. Our survey suggests, that establishing EU level guidelines for screening would be useful, although they would have to take into account differences between individual countries.

## 1. Introduction

Since the early 1990s, there has been a marked rise in migration in Europe, and it is estimated that migrants now constitute 6%–9% of the total European population [1]. European Centre for Disease Prevention and Control (ECDC) stated in 2009, that more than 70% of the estimated 25 million foreigners living in the European Union (EU) come from Eastern and South-eastern Europe and North Africa, and there is diversity also in their reasons of migration [2]. Additionally, also due to geopolitical reasons, some EU countries experience the effects of periodic exceptional inflows of new economic immigrants or of asylum seekers, such as in the case of southern EU countries after the 2011 Arab Spring [3,4].

The changes in migration dynamics have raised concern on the potential effect of migration on the transmission of infectious diseases in the EU, and hence on public health in the region. Migrants in the EU are a highly heterogeneous population, consisting of refugees, asylum-seekers, family reunifications and economic migrants, all with different socio-economic backgrounds, originating from areas where the prevalence of infectious diseases and standards of healthcare might be different compared with their European host countries. There is evidence that these demographical changes may contribute to the burden of infectious diseases, such as tuberculosis (TB) and hepatitis B, in countries receiving immigrants [5,6,7]. There are also examples of migration affecting the spread of human to human transmitted infections, and the risk for reintroduction of vector borne diseases in Europe [8,9,10]. 

In the EU, information on infectious diseases among migrants remains patchy, lacking comprehensive and continuous data. Health information systems in most European countries are generally not designed to collect migrant-specific variables such as migration status, and they often cannot reach people who are not seeking healthcare, because of langue barriers or different societal and cultural factors, not to mention migrants without a regular status [5,11]. In these cases, or when considering diseases that might remain asymptomatic for longer periods, screening newly arrived migrants for infectious diseases could be a useful tool to further monitor their health, and for identifying new or asymptomatic cases of an infectious disease, although reaching migrants without a regular status still remains a problem. Screening can also offer opportunities for prevention and early detection of a disease, for example in the case of TB where identifying latent infection is valuable to prevent later adverse health effects on the individual level [12]. At the same time, it should be noted that screening must be adequately conducted in order to avoid potential harm, such as social stigma or discrimination.

In general, some countries have implemented screening programmes also among newly arrived migrants, especially for tuberculosis (TB) [13,14]. These screening programmes for migrants can be linked to treatment programmes and can benefit individuals, but they can also have a specific public health purpose in helping to assess the burden of a disease and in identifying public health risks, as for example in the immigration medical examinations performed in Canada [15]. However, the information on current screening programmes and practices in Europe is limited, and the factors influencing the differences in chosen practices are not clear.

In order to further develop the surveillance and monitoring of infectious diseases among migrant populations in the EU, our aim was to establish the extent to which countries have implemented screening programmes targeted for newly arrived migrants. We also investigated if the implementation of screening programmes reflects the rate of asylum-seekers in the population in the last five years, asylum-seekers representing one subgroup of all the newly arrived migrants for whom data was available. In addition, our aim was to determine which diseases are routinely screened for, where and for which target populations the screening programmes are implemented, and what is their usefulness.

## 2. Materials and Methods

### 2.1. Questionnaire and Definitions

We developed a 15-point questionnaire on screening among newly arrived migrants in EU/EEA (European Economic Area) countries and Switzerland. The questionnaire was sent electronically by using a web-based survey tool to 28 country experts in March 2014 [16]. Each country expert was officially nominated by the National Competent Body for infectious diseases to take part in an ECDC expert meeting on the Public health benefits of screening for infectious diseases among newly arrived migrants to the EU/EEA held in Athens in 19–20th March 2014. Those who did not reply to the questionnaire after the initial contact, were sent a first reminder two weeks later, and a second reminder was given during the ECDC expert meeting.

For the questionnaire, screening was defined as a systematic practice of medical examination, involving laboratory and/or other diagnostic testing, for searching and identifying cases of a specific infectious disease in a target population. Newly arrived migrants, adapting the UN definition of migrants, were defined as persons, other than travelers or tourists, who had arrived in the last year (less than 12 months) to a country other than their usual residence [17].

The questionnaire investigated the current screening practices and guidelines in each EU/EEA country, both at national and sub-national level. For each implemented screening programme, respondents were asked to specify the diseases screened for, at what level in the migration process screening took place, what was the target population, and whether the screening was compulsory for these target populations. More than one level and target population could be indicated. The different levels for screening were defined as (I) pre-entry level, screening before entering or travelling to the receiving country; (II) entry level, screening at the point of entry (e.g., harbours or airports) (III) holding level, screening in the migrant centres defined as reception/holding/transit facilities commonly used to house asylum-seekers; or (IV) community level, screening after arrival and after partial integration to the community in the receiving country (e.g., in the primary care) [13]. Potential target populations for screening were defined as (I) all newly arriving migrants; (II) asylum-seekers; (III) arrivals from endemic areas or (IV) other target groups, with a possibility to further specify.

We also asked the respondents to describe whether the screening data collected from their implemented screening programmes was generally available for public health purposes, and what actions, such as vaccination campaigns, treatment or control measures, were taken based on the screening results. Finally, we asked their expert opinions on the general usefulness of screening programmes, what is their opinion on the screening programmes implemented in their countries, and what are their opinions on the perceived need to establish EU level guidelines for screening, or to provide other support on screening targeted for newly arrived migrants. 

The study does not report any experiment on human or biological human samples, nor research on identifiable human material and data, it is an observational survey based on National Expert’s professional opinions in the field of migrant screening.

### 2.2. Analysis

Furthermore, we acquired data on asylum-seekers and resident populations from EUROSTAT between the years 2008 and 2013. We chose the data on asylum-seekers as a proxy for all newly arrived migrants, as in the available information on migrant flows the migrants were not defined as having arrived during the last year, and therefore did not meet our definition for newly arrived migrants. We calculated the mean number of asylum-seekers per year between 2008 and 2013, and divided the number of asylum-seekers by the number of the resident population in the middle of the period. Based on this mean rate of asylum-seekers in the population 2008–2013 we ranked the countries and further categorized them into three groups of equal size: low (<16/100,000), medium (16–92/100,000) and high (>92/100,000) rate of asylum-seekers in the population. We studied the association between this parameter and the implementation of screening programmes and national guidelines by using a chi-squared test.

The data were analyzed by using STATA version 11.0 [18]. A frequency analysis was performed for all the categorical variables, and the proportions of responses were summarized. The chi-squared test was used for testing differences in frequency of categorical variables.

## 3. Results

Of the 28 country experts enrolled, 27 (96%) submitted a valid completed questionnaire. All the respondents were infectious disease experts from national institutes of public health or from national ministries of health, from 27 different EU/EEA countries.

Screening among newly arrived migrants was implemented in 59% (16/27) of the responding countries. National guidelines for screening among newly arrived migrants, at least for one disease, were available in 56% (15/27) of the countries. Forty-eight percent (13/27) of the countries had both implemented screening programmes and guidelines for screening; the implementation of screening and the existence of national guidelines was associated (*p* = 0.001).

When comparing the rate of asylum-seekers in the population and the implementation of screening programmes, countries with high rate had implemented screening programmes more often (Table 1). Similarly, those with high or medium rate of asylum-seekers, had more often guidelines for screening among newly arrived migrants. Having a high rate of asylum-seekers in the last five years was associated with the implementation of screening programmes (*p* = 0.014), as well as with the existence of relevant guidelines (*p* = 0.005).

**Table 1:** Association between the rate of asylum-seekers in the population and the implementation of screening programmes and guidelines for screening.

	Low Rate	Medium Rate	High Rate	*p*-value
Countries with implemented screening programmes	22% (2/9)	67% (6/9)	89% (8/9)	0.014
Countries with guidelines for screening	11% (1/9)	78% (7/9)	78% (7/9)	0.005

**Figure 1:**
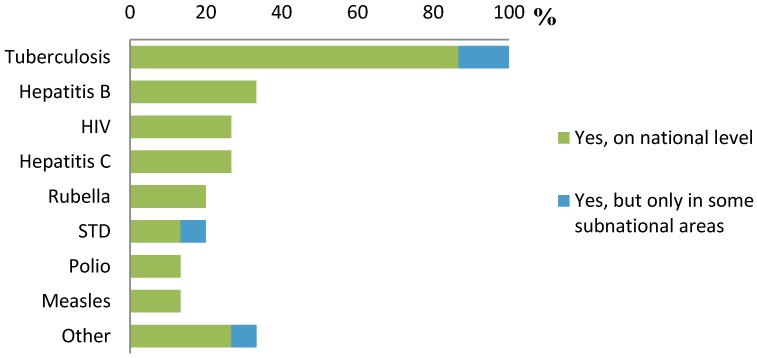
Infectious diseases screened for in countries with implemented screening programmes (*n* = 15).

By far, the most common disease screened for was TB (Figure 1). All experts that reported having implemented routine screening programmes and who responded to the question on specific diseases screened for (*n* = 15), reported screening for TB. Other diseases screened for included hepatitis B (5/15, 33%), hepatitis C (4/15, 27%) and HIV (4/15, 27%). Sexually transmitted diseases (STD) and vaccine preventable diseases (VPD) were screened for in 3/15 (20%) of the countries, and 33% of the experts reported screening activities in their country for other diseases (e.g., cholera, malaria). In total, 9/15 (60%) of the countries screened for diseases other than TB.

**Figure 2:**
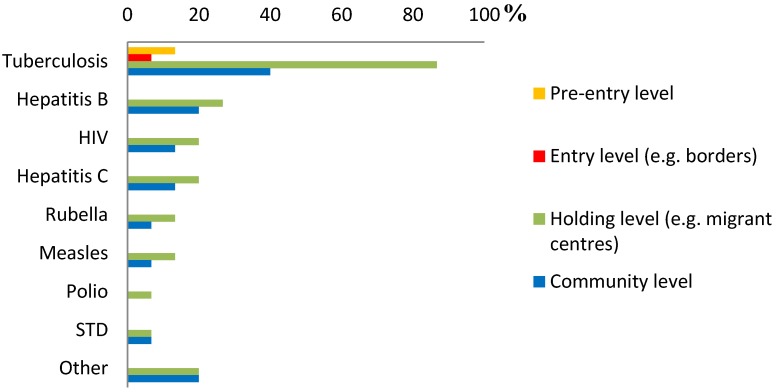
The level for screening for each infectious disease in countries with implemented screening programmes (*n* = 15).

Overall, screening was most commonly performed at the holding level, followed by screening at the community level after arrival (possibly more than one level for each disease) (Figure 2). For TB, screening at the holding level was most common: 87% of respondents reported screening at the holding level and 40% at the community level. Pre-entry screening was reported to be implemented only by two countries (13%) and entry level screening by one country (7%), and both only in the case of TB. For Hepatitis B, 27% reported screening at the holding level and 20% at the community level. For hepatitis C, 20% reported screening at the holding level and 13% at the community level. For HIV, 20% reported screening at the holding level and 13% at the community level. For other diseases the level of screening varied similarly.

Sixty-nine per cent (11/16) of experts, reporting implemented screening programmes in their country, responded that their screening programmes were targeted to one or more subgroups of newly arrived migrants. Target populations included: asylum seekers (11/16, 69%); arrivals from endemic areas (7/16, 44%); other target groups (e.g., children or specific subgroups of asylum-seekers) (6/16, 38%). Screening was reportedly performed among all newly arrived migrants without further target groups in 25% (4/16) of the countries performing screening, and it was compulsory for each target group in 60% (9/15) of the countries performing screening. 

The data on screening was collected and available for public health purposes in 64% (9/14) of the countries. The actions directed by these data included treatment in the case of disease detection (80%, 12/15), improvement of the access to the national health care systems (73%, 11/15), pre/post-screening counselling (73%, 11/15), providing international health authorities information of possible public health threats (67%, 10/15), isolation or other control measures (53%, 8/15) and vaccination campaigns (47%, 7/15).

**Figure 3:**
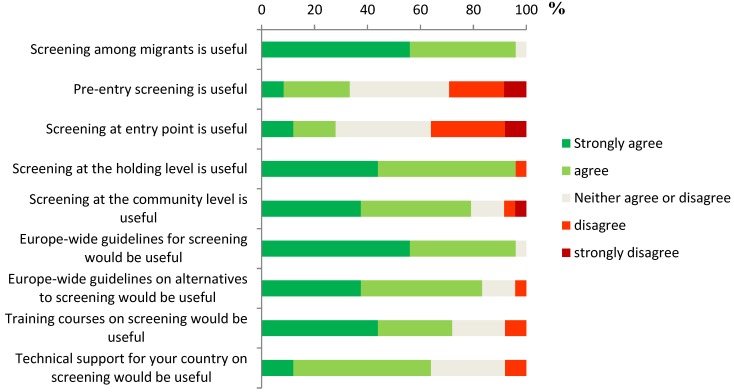
Expert opinions on the usefulness of screening and of potential actions taken on international level (*n* = 26).

Screening among migrants was considered useful by 96% (25/26) of the country experts participating to the survey and responding the question; especially if the screening was conducted at the holding level (25/26, 96%) and at the community level (20/26, 77%) (Figure 3). Pre-entry level screening was considered useful by 31% (8/26) of the respondents, and similarly, 27% (7/26) considered entry-level screening useful. In total, 21% of the respondents considered that the screening was well structured and 24% considered it to be well carried out in their country. Respondents also considered establishing EU level guidelines for screening useful (92%, 24/26). Presenting EU level guidelines for alternatives on screening, or organizing EU level training courses or technical support for the national implementation of screening programmes were also considered useful, but to a lesser extent (77%, 73% and 62%, respectively). 

## 4. Discussion and Conclusions 

At the time of the survey, just over half of the EU/EEA countries had implemented national or sub-national screening programmes targeting newly arrived migrants. The implementation of screening, often in place in migrant centres, was associated with the rate of asylum-seekers in the population. In those countries where screening programmes were implemented, the practices varied, although most of the countries had established national guidelines for screening. Experts participating in the survey widely agreed on the usefulness of screening programmes among newly arrived migrants and on the need to establish EU level guidelines on this issue. 

Our study shows that countries with screening programmes targeting newly arrived migrants did so first and foremost to detect TB, confirming previous findings of international studies on screening programmes [13,14]. Screening for other infectious diseases was implemented in one third of all EU/EEA countries, but diseases screened for varied by country. Screening programmes were specifically targeted for subgroups of newly arrived migrants, most often asylum-seekers at the holding level. Also, other subgroups were targeted in some countries, but this depended on the disease in question and on the availability of resources. 

Certain studies on screening have proved that it can be reasonably cost-effective and possibly useful in helping to reduce the burden of the disease for infections such as TB or Hepatitis B, although there remains further discussion on how, where and for who screening should be implemented and where it is most effective [19,20,21,22]. For example, in the case of TB, especially certain vulnerable populations are thought to benefit from screening, as difficult travel and housing conditions increase the risk of the disease, thus making screening on the holding level useful [23]. However, in practice all newly arrived migrants cannot be automatically reached in migrants centres, and in some cases diseases can develop years after the migration, and sometimes they can only be detected in later phases. Therefore, depending on the disease, it can be also reasonable and appropriate to perform screening at the first contact with the healthcare, *i.e.*, often at the community level after the arrival, and similarly the importance for screening for certain latent conditions is highlighted to be able prevent later adverse health effects of diseases such as TB or HIV [12,19,21].

As noted above, many of the countries that had implemented screening programmes, had chosen to perform screening commonly in migrant centres that are used to house asylum-seekers, and asylum-seekers are, respectively, the most common target group for screening. These choices may reflect certain practical aspects of implementing screening programmes, but may also be based on certain assumptions of usefulness of screening in settings such as migrant centres.

While taking into account that screening programmes for newly arrived migrants were different, and implemented at different levels, the overall perception of the experts on the usefulness of screening targeting newly arrived migrants was clear, especially at the holding level. Additionally, a variety of actions were taken on the basis of screening results, and in majority of countries treatment was provided in the case of disease detection, indicating that screening is often linked with a treatment programme. These actions support the idea that screening programmes and their results can provide useful information to guide public health actions and are thus valuable tool for monitoring infectious diseases among migrants. However, although screening programmes were considered useful, only less than a quarter of the country experts thought that screening was well structured or well carried out in their respective countries, which indicates that there is a further need for guidance and room for improvement in the currently implemented programmes.

Our survey has certain limitations. First of all, the national practices were inquired from nominated focal points and their expert opinion was asked. In fact, the answers of the experts may not in all cases take into consideration that different areas within countries might well have practices that are not adopted nationally, especially in certain countries. It is also clear that guidelines for screening do not often exist for screening as such, but are specially established for certain prioritized diseases, which makes the question on whether there are guidelines a very difficult one to answer.

Another potential limitation in our study is that we did not ask the differences in practices between individual prioritized diseases, and our definition of screening was very broad. Whilst including all infectious diseases, and various screening methods into our survey we reached a scope of assessing screening practices on a very broad scale, we also lost the specificity of information on methods and other details of each screening programme, targeted for commonly screened diseases, such as TB or hepatitis B. Furthermore, our analysis on the rate of asylum-seekers in the population took into consideration only one subgroup of newly arrived migrants, but did not take into account other important migrant groups, such as family reunifications or resettled refugees, or total migration, for which data on new arrivals was not available. Therefore, our analysis could only give a partial view on the situation in EU/EEA countries. Also, our survey was only able to reach practices implemented for regular migrants; illegal migration remains a challenge that could not be taken into account in our analysis. 

However, our study did give a first picture of the current screening practices for different infectious diseases in EU/EEA countries, and it gave information on the expert opinions on screening from 27 different European countries. Our analysis also took into account the rate of asylum-seekers in the population, which showed an association between a high rate of asylum-seekers in the population and the implementation of screening programmes or national guidelines. Although this does not mean that public health policies have thoroughly influenced the chosen screening practices, as the study only evaluated the association between the current practices and one important immigrant group in the last five years, it does suggest that screening is seen as a potentially good practice especially in countries who receive the highest number of asylum-seekers. This seems to be also reflected in the chosen target groups and the chosen level of screening (migrant centres), as well as in the considerations of usefulness of screening. However, also other target groups such as arrivals from endemic areas could be considered for certain conditions, as is already done in certain countries [14].

As the EU/EEA country experts considered screening among newly arrived migrants useful, and as they would welcome EU level guidelines for screening, establishing such guidelines is recommended. These international guidelines on screening among migrants should however take into account also the differences between countries receiving immigrants, the number of arriving migrants and whether they were refugees, asylum-seekers or economic migrants. Also, considerations on the usefulness and the level or other practices of screening are important to note in the development of the guidelines, to better enhance the potential public health benefits provided by the screening programmes.

Whether screening among migrants for infectious diseases is effective or cost-effective from a public health perspective, remains an open question in our study and needs further research. Literature does suggest that in the cases of TB and hepatitis B screening can be maintained on a relatively effective basis, and further consideration on the effectiveness of screening programmes is clearly important for the development of guidelines [20,21]. But it is also clear, that screening is not to be seen only as a tool for cost-effectiveness of health care, but also as a tool for improving the situation of vulnerable populations, and it could be simply considered as a part of routine healthcare in most of the immigrant subgroups [24].

When discussing the benefits of screening, the importance of linking screening with disease-specific treatment programmes, or using the screening-data for public health purposes (e.g., by organizing vaccination campaigns) is clear, and such activities already take place in majority of countries performing screening. This is a clear sign of the usefulness of screening programmes, as activities are implemented following the screening, and shows the importance and possibilities of screening, also reminding that screening is not to be seen as a tool for excluding migrants, nor should it even be considered as a tool for such a practice, but a tool to improve the health of migrants for the greater benefit of all.
